# Diagnosis of Local Recurrence of Malignant Soft Tissue Tumors after Reconstructive Surgery on MRI

**DOI:** 10.3390/jcm12134369

**Published:** 2023-06-29

**Authors:** Eun-Hee Song, So-Yeon Lee, Seungeun Lee, Joon-Yong Jung, Seung-Han Shin, Yang-Guk Chung, Chan-Kwon Jung

**Affiliations:** 1 Department of Radiology, Seoul St. Mary’s Hospital, College of Medicine, The Catholic University of Korea, 222 Banpo-daero, Seocho-gu, Seoul 06591, Republic of Korea; 2 Department of Radiology, Kangbuk Samsung Hospital, Sungkyunkwan University School of Medicine, Seoul 03181, Republic of Korea; 3 Department of Orthopedic Surgery, Seoul St. Mary’s Hospital, College of Medicine, The Catholic University of Korea, 222 Banpo-daero, Seocho-gu, Seoul 06591, Republic of Korea; 4 Department of Pathology, Seoul St. Mary’s Hospital, College of Medicine, The Catholic University of Korea, 222 Banpo-daero, Seocho-gu, Seoul 06591, Republic of Korea

**Keywords:** magnetic resonance imaging, reconstructive surgical procedures, recurrence, soft tissue neoplasms

## Abstract

Purpose: Magnetic resonance imaging (MRI) is useful in the diagnosis of local recurrence, but few studies have explored recurrence in MRI in patients after reconstructive surgery. The purpose of this study was to analyze MRI findings of locoregional recurrence following reconstructive surgery after malignant soft tissue tumor resection. Method: Fifty-three postoperative MRIs from 37 patients who underwent reconstructive surgery after malignant soft tissue tumor resection were retrospectively reviewed. A total of 76 enhancing lesions, including 40 locoregional recurrences and 36 postoperative changes, were analyzed regarding morphology (location on the transplanted tissue, border, and shape) and the signals on T1- and T2-weighted imaging (T1WI, T2WI), fat-suppressed (FS) T2WI, and contrast-enhanced FS T1WI. Diffusion-weighted imaging with an apparent diffusion coefficient was assessed. A chi-squared test and Fisher’s exact test were used for statistical analysis. Results: The most common site of recurrent tumors and postoperative changes was the peripheral margin on transplanted tissue (63% and 61%, respectively *p* = 0.907). Recurrent tumors commonly appeared with well-defined borders (75%) as well as nodular appearance (98%), hyperintensity on T2WI (85%) and FS-T2WI (95%), isointensity on T1WI (65%), impeded water diffusion (55%), and intense (50%) or moderate (45%) enhancement. Postoperative changes showed ill-defined borders (75%), nodular appearance (56%), facilitated water diffusion (69%), and moderate (86%) enhancement, which were significantly different from those of recurrent tumors (*p* ≤ 0.020). Conclusions: Common and partitioning MRI features of locoregional recurrence were well-defined borders, nodular shape, impeded water diffusion, and intense enhancement. Peripheral margins on transplanted tissue were common sites in both recurrent tumors and postoperative changes.

## 1. Introduction

Malignant soft tissue tumors of the trunk and extremities include soft tissue sarcomas, melanoma, non-melanoma skin cancers, and metastases [1]. Skin cancer is the most common type of cancer worldwide [2]. In contrast, malignant soft tissue sarcomas are uncommon and account for less than 1% of all malignancies [3,4]. Radical surgical resection with a wide curative margin is the most important therapeutic option for such cancers [4,5]. According to advances in multimodal therapy, including adjuvant chemotherapy and radiation therapy, limb salvage surgery has replaced amputation as the standard treatment [4].

Because of the wide spread of soft tissue defects and the associated functional impairments, reconstructive surgery is often needed. Reconstructive surgery provides stable soft tissue coverage and restores the contours and functioning of the limb. Flaps with strong vascularization offer speedier wound healing and improve the oncologic outcome of sarcoma resection [4]. In addition, it contributes to improving the quality of life of patients through functional recovery and cosmetic improvement. After determining the extent of resection through a multidisciplinary approach, reconstructive surgery is tailored to the patient to address tissue defects. A surgical plan is created based on the presence of previously irradiated tissue, future radiation plans, and wounds that require auxiliary treatment. Magnetic resonance imaging (MRI) both before and after surgery is essential. After soft tissue tumor surgery, there may be large soft tissue deficits, exposure of major blood vessels and nerves, and flap surgery is often necessary considering the function of the joint. Planning of reconstructive surgery should however not be limited to covering defects. The reconstruction method and the type of flap are determined in consideration of the degree, location, and anatomical relationship of the defect. Functional and cosmetic aspects should be considered as well as possible aftereffects from donor sites. There are various methods of reconstructive surgery depending on the type and location of the soft tissue to be transplanted [6]. A skin graft is transplanted without blood supply, whereas a flap is transplanted with blood supply. Flaps can use a larger amount of tissue, including muscles, if required. Flap surgery is classified into two types according to whether they are detached from the blood supply at the original location: free flap and locoregional flap. In the free flap procedure, detached vessels are transplanted to another location, and the circulation in the tissue is reconstructed via anastomosis of the vessels. In the locoregional flap, transplanted tissues are simply transposed to a new location, keeping the vascular supply from the “donor site”. Postoperative complications of reconstructive surgery include total flap loss, thrombosis, and partial necrosis, as well as hematoma, abscess, and wound dehiscence. Reported rates of total flap loss, thrombosis, and partial necrosis were about 6% [7]. In reconstructive surgery, the resected areas are altered for the transplanted autologous tissue, which can cause confusion to clinicians and radiologists as to where local recurrences will develop [4]. Postoperative complications such as hematoma or abscess can also mimic recurrent tumors.

Local recurrence has been reported in up to 10% of patients with cutaneous melanoma [8] and 7–15% of patients with soft tissue sarcoma of the trunk or extremities despite treatment using a specialized multidisciplinary team approach [9,10,11,12]. MRI is routinely used for surveillance of local recurrence in patients who have undergone oncologic surgery. When the reconstructed tissue is thick (e.g., musculocutaneous flaps), it is difficult to detect recurrence through inspection and palpation, so MRI is the best option for detecting recurrence early. In thin reconstructed areas, such as skin grafts, physicians can detect many local recurrences through visual examination and manual inspection. In those cases, the role of MRI is to distinguish recurrence from complications and postoperative changes, as well as to evaluate recurrent tumor extent, rather than detection.

However, diagnosing local recurrence using MRI is often difficult, because postoperative inflammation and fibrosis may show similar characteristics with local recurrence, such as T2 hyperintensity, architectural distortion, and enhancing nodules in the surgical bed [13,14]. In particular, patients who have undergone reconstructive surgery demonstrate anatomical alterations and post-radiation changes in soft tissues, hindering detection of recurrence. Normal imaging findings after reconstructive surgery were reported only as inserted figures in previous studies, but there is no original research on this. In addition, no studies have investigated the MRI features of local recurrence of malignant soft tissue tumor resection after soft tissue reconstructive surgery [5].

Therefore, the purpose of our study was to analyze MRI findings of benign postoperative changes and locoregional recurrence following reconstructive surgery after tumor resection. We also hypothesized that some characteristic MRI features can be used to differentiate recurrence from postoperative changes in patients who undergo tumor resection and reconstructive surgery. Furthermore, we sought to identify distinguishing MRI features of local recurrence.

## 2. Methods and Materials

### 2.1. Study Design

This retrospective study was approved and informed consent was waived by the institutional review board (KC21RISI1019, Seoul St. Mary’s Hospital Institutional Review Board, approved date: 7 January 2022). We retrospectively compared postoperative MRI findings of patients who had undergone soft tissue sarcoma resection and reconstructive surgery after dividing them into two groups—those with and without recurrence. The definition of the group with recurrence was patients who had undergone biopsy to confirm recurrence for focal abnormal findings in postoperative MRI. The definition of the group without recurrence was patients who had pathologically or clinically confirmed benign lesions that had appeared as focal abnormal findings in postoperative MRI.

### 2.2. Patients

**Figure 1:**
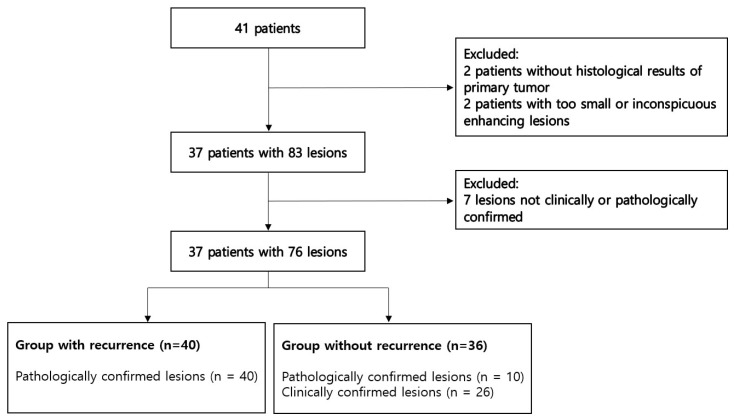
Flow chart of patient inclusion.

To divide patients into the two study groups (with and without recurrence), we reviewed medical records including operation records, pathology reports, and multidisciplinary case conference records. Postoperative MR images after tumor excision and reconstructive surgery were also reviewed. Postoperative MRI findings refer to follow-up MRI scans taken on a set schedule after reconstructive surgery, usually every 3–6 months during the first 1–2 years, depending on the histologic type and grade. We selected patients and lesions using the following inclusion and exclusion criteria. Inclusion criteria were: ① adult (≥17 years) patients who had undergone malignant soft tissue tumor resection followed by soft tissue reconstruction between 1 January 2010 to 31 October 2021 in our institution; ② focal enhancing lesion in any location of the scanned surgical site in postoperative MRI; and ③ lesion met either (a) or (b)–(a) pathologically confirmed lesion using excisional or incisional biopsy or image-guided core needle biopsy, (b) clinically confirmed benign lesion defined as a lesion with a >30% decrease in longest axis measurement during follow-up MRI after at least 1 year without adjuvant chemotherapy or radiation therapy. Exclusion criteria were: ① patients without histological results regarding resected malignant soft tissue tumor; ② too small (<1 cm) or inconspicuous enhancing lesions in postoperative MRI that were defined as not clearly visible due to the weak degree of contrast enhancement and thus not described in the MRI report; ③ subtle linear enhancing lesions along the surgical scar presumed to be postoperative changes, observed clinically or radiologically; and ④ pathologically unidentified lesion that had undergone adjuvant chemotherapy or radiation therapy. When the patient underwent several MRI exams without additional operation, only the exam on which the lesion(s) were seen foremost was selected. Follow-up MRI after additional reconstructed surgery was separately included. For example, one patient underwent MRI 3, 6, 9, 12, and 18 months after reconstructive surgery. This patient underwent re-operation at 13 months to resect a recurrent tumor and also underwent a new reconstructive operation. In this case, the study included one 12th month MRI with the most distinct lesions among the 3rd month, 6th month, and 12th month MRIs. In the case of the 18th month MRI, an 18th month MRI is also included in this study, considering that it is a new case because a new reconstructive operation was performed. Selecting MRI in this way involves newly detected or enlarged lesions during follow-up in an actual clinical setting. When there were multiple enhancing lesions in one MRI, the lesions were separately included as recurrence or postoperative changes according to each pathology result. Included enhancing lesions were classified as a recurrent tumor or benign postoperative change such as inflammation or fibrosis. Finally, 40 local recurrences and 36 postoperative changes were included based on the inclusion and exclusion criteria (Figure 1).

We extracted patient demographic data from medical records, including age, sex, anatomic localization of tumor, type of reconstructed surgery (free flap, local flap, skin graft), and history of previous radiation therapy. Histologic tumor types of primary tumors and recurred tumors and the presence of tumor cell infiltration in the surgical margins of excised primary tumors were also obtained from pathologic reports. We calculated the time span between reconstructive surgery and the postoperative MRI. We also calculated the time span between the postoperative MRI and reoperation. If the patients underwent fluorine-18-fluorodeoxyglucose positron emission tomography/computed tomography (18F-FDG PET/CT) examination, we collected the maximum standardized uptake values (SUV_max_) of the lesions.

### 2.3. MRI Protocol

MRI was performed using one of three 3T MRI scanners (Magnetom Verio; Siemens Healthineers; Erlangen, Germany) with dedicated surface coils according to the anatomical site. The standard MRI protocols included various sequences: longitudinal fat-suppressed (FS) turbo spin-echo (TSE) T2-weighted image (T2WI), axial TSE T1-weighted image (T1WI), T2WI with and without FS, and longitudinal and axial FS contrast-enhanced T1WI. Other parameters are described in Table 1. Single-shot spin-echo echo planar diffusion-weighted MRI (DWI) was obtained in the axial plane before contrast enhancement. A parallel imaging technique using GRAPPA (GeneRalized Autocalibrating Partially Parallel Acquisitions) was conjugated with an acceleration factor of 2. With *b*-values of 0 and 800 s/mm^2^, sensitizing diffusion gradients were applied sequentially in the *x*, *y*, and *z* directions. As a basis of mono-exponential calculation from DWI, pixel-based apparent diffusion coefficient (ADC) maps were made using commercial software and a workstation (Leonardo MR Workplace; Siemens Healthineers) [15].

### 2.4. MRI Interpretation

**Figure 2:**
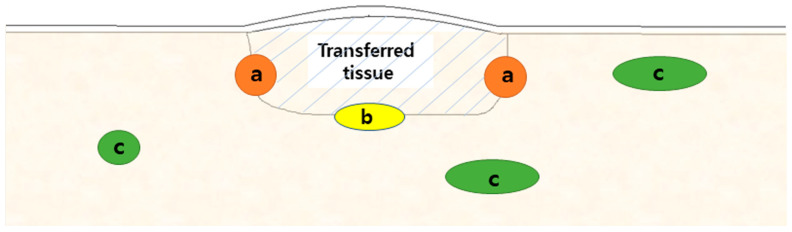
Schematic drawing of enhancing lesions on transplanted tissue: (a) peripheral margin, (b) deep margin, and (c) regional location.

Image analysis was performed by two board-certified radiologists (S.-Y.L., 13 years of experience in musculoskeletal imaging; E.-H.S. with 7 years of experience in general imaging) in consensus. For avoiding recall bias, MRIs were reviewed after 8 weeks from lesion selection in MRI. Reviewers were blinded to clinical information. The 76 enhancing lesions were evaluated according to morphology, signal intensity in each MRI sequence, and the presence of impeded water diffusion as follows: ① location associated with transplanted soft tissue margin (peripheral margin, deep margin, regional location, which is more than 2 cm apart from the surgical margin) (Figure 2) [16]; ② border (well-defined, ill-defined); ③ shape (nodular, band-like, diffuse, spiculated); ④ maximum diameter measured on axial FS contrast-enhanced T1WI; ⑤ signals intensities of lesions on T1WI, T2WI, FS-T2WI, DWI with *b*-value of 800 s/mm^2^ (hyperintense, isointense, hypointense) as compared with those of muscles on the same image planes; ⑥ ADC value measured by manually drawn regions of interest (ROI) on the ADC map on a picture archiving and communication system (PACS); the ROI were placed over the most enhanced solid portions. The borders of the enhanced lesion were not included to avoid partial volume artifacts, and did not include necrotic or cystic areas; ⑦ presence of impeded water diffusion, which was defined as hyperintensity on DWI with *b*-values of 800 s/mm^2^ and low value on corresponding ADC maps [15]; and ⑧ contrast enhancement following IV contrast administration (intense, moderate, mild) as compared with those of vessels and muscles on the same imaging planes. ‘Intense’ indicates intensity similar to blood vessels, ‘moderate’ indicates brightness that is stronger than muscle and weaker than blood vessels, and ‘mild’ means brightness similar to or weaker than muscle.

### 2.5. Statistical Analysis

MRI parameters were compared between recurrence and postoperative changes using an independent *t*-test for continuous variables and chi-square test and Fisher’s exact test for categorical variables. *p* ≤ 0.05 was considered indicative of a statistically significant difference. The sensitivity, specificity, accuracy, and positive and negative predictive values were calculated using pathologic and clinical results as the gold standard. Statistical analyses were performed using commercially available software (MedCalc^®^ Statistical Software version 20.106 (MedCalc Software Ltd., Ostend, Belgium; https://www.medcalc.org; 21 May 2022)). Stratified statistical analysis was performed after controlling for potential confounders with the Mantel–Haenszel test using another commercially available software (IBM Corp. Released 2016. IBM SPSS Statistics for Windows, Version 24.0. Armonk, NY, USA; IBM Corp.; https://www.ibm.com; 21 May 2022).

## 3. Results

### 3.1. Characteristics of the Included Patients and Enhancing Lesions

**Figure 3:**
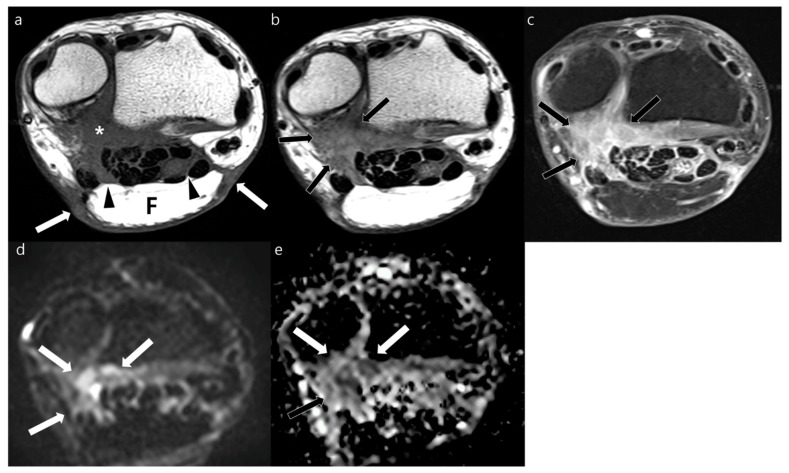
Postoperative MRI obtained at the wrist joint of a 31-year-old woman who underwent planned R1 surgical resection of pseudomyogenic hemangioendothelioma with immediate free flap reconstruction 3 months prior. (**a**) Axial T1WI shows flap (F) with peripheral (arrows) and deep (arrowheads) margins in the volar side of flexor tendons. An isointense lesion (asterisk) is abutting to the deep margin of the transplanted tissue. (**b**) Axial T2WI shows ill-defined nodular hyperintense lesions along deep margin (arrows). (**c**) Axial contrast-enhanced FS-T1WI shows moderate enhancement (arrows). (**d**,**e**) This lesion (arrows) represents impeded water diffusion seen as hyperintense signal on axial DWI with a *b*-value of 800 s/mm^2^ (**d**) and low ADC (**e**). These lesions were confirmed as recurrence of pseudomyogenic hemangioendothelioma via excisional biopsy.

**Figure 4:**
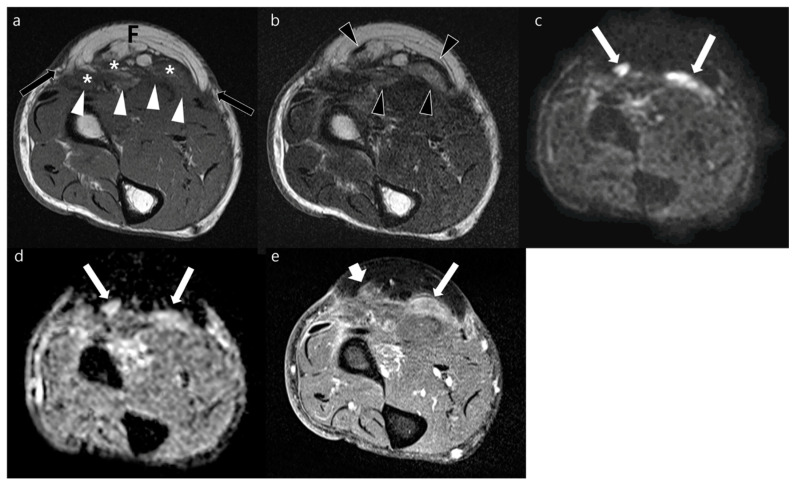
Postoperative MRI obtained from the forearm of a 77-year-old man who underwent surgical resection of myxofibrosarcoma with immediate free flap reconstruction in the forearm 6 months prior. (**a**) The axial T1WI shows flap (F) with peripheral (arrows) and deep (arrowheads) margins in the volar side of forearm. Ill-defined, heterogeneous lesions (asterisks) are seen along deep margin of the transplanted tissue. (**b**) Axial T2WI shows ill-defined nodular hyperintense lesions (arrowheads). (**c**,**d**) These lesions (arrows) represent facilitated water diffusion seen as hyperintense signal on axial DWI with a *b*-value of 800 s/mm^2^ (**c**) and high ADC (**d**). (**e**) Axial contrast-enhanced FS-T1WI shows focal enhancement of moderate (arrow) and mild (short arrow) degrees. These lesions decreased in size and finally disappeared in enhancement in serial follow-up MRIs without other treatment; they were then presumed as postoperative changes.

**Figure 5:**
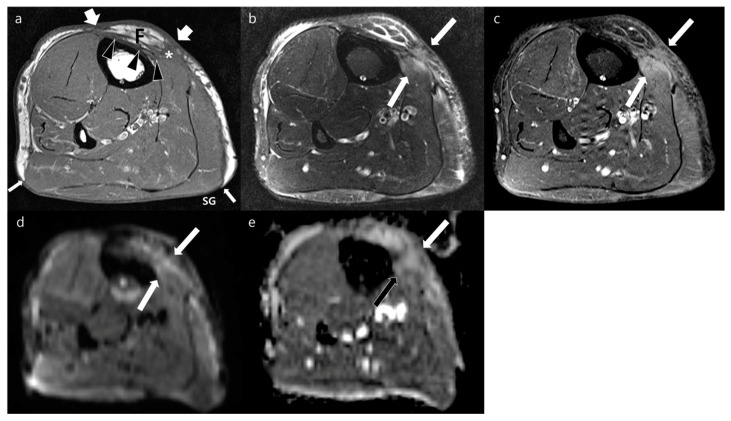
Postoperative MRI obtained from the leg of a 46-year-old woman who underwent surgical resection of myxofibrosarcoma with immediate local flap reconstruction at the anterior tibial region 6 months prior. (**a**) Axial T1WI shows a flap (F) with peripheral (arrows) and deep (arrowheads) margins on the anteromedial side of the leg. Ill-defined isointense lesion (asterisk) involved deep and peripheral margins of the transplanted tissue. The skin graft (SG) is noted as a thin hypointensity overlying the gastrocnemius muscle. The peripheral margin of the skin graft is indicated by thin arrows. (**b**) Axial T2WI shows ill-defined nodular hyperintense lesion (arrows). (**c**) Axial contrast-enhanced FS-T1WI shows moderate enhancement (arrows). (**d**,**e**) This lesion (arrows) represents facilitated water diffusion seen as hyperintense signal on axial DWI with a *b*-value of 800 s/mm^2^ (**d**) and high ADC (**e**). This lesion decreased in size in follow-up MRI without other treatment; it was then presumed as a postoperative change.

**Figure 6:**
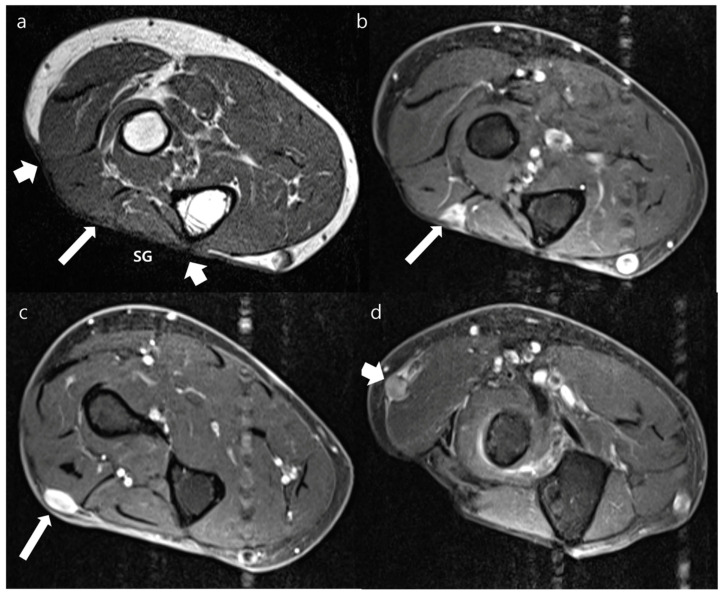
Postoperative MRI obtained from the forearm of an 88-year-old woman who underwent surgical resection of myxoid liposarcoma with immediate skin graft at the forearm 4 years prior. (**a**) The axial T1WI shows skin graft (SG) with peripheral (short arrows) margins on the dorsal side of forearm. An isointense nodule involves the deep margin of the transplanted tissue (arrow), which was not well delineated because of similar signal intensity with that of perilesional muscle. (**b**) Axial contrast-enhanced FS-T1WI obtained at the same section with (**a**) shows moderate enhancement of lesion (arrow) at the deep margin of SG. (**c**) Axial contrast-enhanced FS-T1WI obtained 2.4 cm distal to (**a**,**b**) shows another lesion arising from peripheral margin (arrow). This lesion has a well-defined nodular appearance with intense enhancement. (**d**) Axial contrast-enhanced FS-T1WI obtained 2.0 cm proximal to (**a**,**b**) shows another lesion in the regional location, which was apart from transplanted tissue (arrow). This lesion has a well-defined nodular appearance with moderate enhancement. These three lesions were confirmed as recurrence of myxoid liposarcoma via excisional biopsy.

Our study included 37 patients with 19 male patients and 18 female patients. The mean patient age was 60.1 years (range, 31–89 years). The most common histopathology of tumors diagnosed via excision before reconstructive surgery was cutaneous melanoma (*n* = 11 patients) followed by myxofibrosarcoma (*n* = 6 patients) (Table 2). The mean interval between tumor resection with subsequent soft tissue reconstruction and postoperative MRI was 31 months ± 37 (median, 7 months). The mean interval of postoperative MRI and biopsy of enhancing lesions on postoperative MRI was 18 days ± 28. The mean duration of follow-up of non-operative lesions was 4 years ± 3. Forty recurrent tumors (Figure 3 and Figure 4) were found in 19 patients, and 36 postoperative changes (Figure 5 and Figure 6) were found in 27 patients. The histopathology and anatomic location of the primary tumor varied (Table 3). Twenty-four lesions were seen at previous R1 resection sites showing presence of a microscopic residual tumor. Three types of reconstruction surgery were performed: free flap using musculocutaneous and fasciocutaneous tissues (*n* = 42), local flap (*n* = 21), and skin graft (*n* = 13). Forty-one lesions were in a post-radiated state. There were 41 lesions that had matching lesions on 18FDG-PET/CT. The mean SUV_max_ was 8.57 ± 9.22 for 22 recurrent lesions and 2.00 ± 2.12 for 19 postoperative lesions.

### 3.2. Morphology of Recurrent Tumors

Recurrent tumors were most commonly present in the peripheral margin (*n* = 25) followed by the deep margin (*n* = 11) on transplanted soft tissue, which was not significantly different from those with postoperative changes (*p* = 0.907, and 0.583, respectively). Seven regional recurrences were seen apart from transplanted tissue. The borders of the lesions were frequently well-defined (30/40, 75%) in recurrent tumors but were ill-defined (27/36, 75%) in postoperative changes (*p* < 0.001). Of recurrent tumors, 98 percent (39/40) were nodular in appearance, whereas of postoperative changes, 56 percent (20/36) were nodular in appearance (*p* < 0.001). One recurrent tumor (undifferentiated pleomorphic sarcoma) showed a band-like appearance. Sixteen postoperative changes showed a non-nodular appearance, including band-like (*n* = 8), diffuse (*n* = 4), and spiculated appearances (*n* = 4). The mean size of the tumors in the recurrence group was 2.6 cm ± 2.2, whereas that of the postoperative change group was 1.7 cm ± 1.0 (*p* = 0.020) (Table 4).

### 3.3. MRI Signal Intensity of Recurrent Tumor

The signal intensity of both local recurrence and postoperative changes were significantly different in each sequence (Table 5). Local recurrences were commonly seen as isointensity (26/40, 65%) in T1WI and hyperintensity on T2WI (34/40, 85%) and FS-T2WI (38/40, 95%). On contrast-enhanced FS-T1WI, local recurrent lesions showed intense (20/40, 50%), moderate (18/40, 45%), and mild (2/40, 5%) enhancement. Local recurrence was commonly seen as hyperintensity on DWI (37/40, 93%) and was not significantly different from that of postoperative changes (30/36, 83%). The mean ADC of local recurrence was significantly lower than that of the postoperative group (1297.0 μm^2^/s ± 590.9 vs. 1634.6 μm^2^/s ± 462.7, *p* = 0.008). Recurrent tumors more commonly showed impeded water diffusion than postoperative changes (22/40 vs. 5/36, *p* < 0.001).

### 3.4. Diagnostic Performance of MRI Features for Diagnosing Local Recurrence

The nodular shape showed high sensitivity and a negative predictive value of 97.5% and 94.1%, respectively. Strong enhancement and impeded water diffusion showed high specificity (91.7% and 86.1%, respectively) and positive predictive values (87.0% and 81.5%, respectively) (Table 6).

### 3.5. Post hoc Analysis

Seven regional recurrent tumors of seven patients were varied in pathology: myxofibrosarcoma (*n* = 3), myxoid liposarcoma (*n* = 2), and melanoma (*n* = 2). Twelve recurrent tumors with hyperintense signal on T1WI of 11 patients were also varied in pathology: myxoid liposarcoma (*n* = 4), myxofibrosarcoma (*n* = 2), melanoma (*n* = 2), undifferentiated pleomorphic sarcoma (*n* = 2), dedifferentiated liposarcoma (*n* = 1), and squamous cell carcinoma (*n* = 1). Hyperintensity on T1WI was observed significantly more frequently in recurrent tumors than postoperative changes after adjustment for whether or not it was melanoma (*p* = 0.019). Hyperintensity on T2WI was observed significantly more frequently in recurrent tumors than in postoperative changes after adjustment for whether or not it was myxoid-containing tumor (*p* = 0.001). Hyperintensity on FS-T2WI was not significantly different between recurrent tumors and postoperative changes after adjustment for whether or not it was a myxoid-containing tumor (*p* = 0.052). The locations of enhancing lesions were not different between recurrent tumors and postoperative changes after adjustment for soft tissue reconstruction type (*p* = 0.984, 0.900, and 0.416 for peripheral margin, deep margin, and regional location, respectively). Signal intensity on T2WI was observed significantly more frequently in recurrent tumors than in postoperative changes after adjustment for whether or not it was a myxoid-containing tumor (*p* = 0.001). Signal intensity on T1WI, T2WI, FS-T2WI, CE, and diffusion restriction were significantly different between recurrent tumors and postoperative changes after adjustment for whether or not they were post-radiation tissues (*p* ≤ 0.047). Well-defined borders and nodular shapes were observed significantly more frequently in recurrent tumors than in postoperative changes after adjustment for whether or not they were post-radiation tissues (*p* ≤ 0.002). Histologically confirmed benign lesions (*n* = 10) were comprised of chronic inflammation and fibrosis with (*n* = 2) and without (*n* = 6) foreign body reaction and fragments of benign nerve bundles (*n* = 2).

## 4. Discussion

This study analyzed MR imaging findings of locoregional recurrence as well as common benign postoperative findings in patients undergoing reconstructive surgery after tumor resection. Since little is known about postoperative MRI findings in such patients, we believe that our results in incidence, shape, and location of postoperative changes can help to increase such knowledge. The principal MRI features of locoregional recurrence were well-defined borders, nodular shape, impeded water diffusion, and intense enhancement. The MRI features suggestive of benign postoperative changes were ill-defined borders, non-nodular shape, and free water diffusion.

Local tumor recurrence was most commonly seen in the peripheral margin of the transplanted tissue followed by the deep margin, regardless of the type of reconstructive surgery in our study. Little is known about locations of local tumor recurrence in patients undergoing reconstructive surgery after tumor resection. Kotnis et al. reported postoperative MRI findings of myocutaneous flap reconstruction following hindquarter amputation for pelvic musculoskeletal malignancy [17]. They showed 23 recurrent tumors in 77 MRI examinations, of which 43% were in the muscle component of the amputation flap, 13% were in subcutaneous tissues of the flap, and 13% were at the posterior margin of the bone resection. Fujiki et al. [4] reported local recurrence of soft tissue sarcoma resection with flap reconstruction in 14.4% of their sarcoma study group; 69% were in the peripheral margin of the transplanted flap; and 31% were in the deep layer of the transplanted flap. Similar incidence was observed in our MRI study. Since small lesion or local recurrence arising from the deep margin may not be palpated upon physical examination, thorough evaluation of the surgical margin in MRI is important. Knowledge of flap margins may increase the detection rate for recurrent tumors. A new finding in our study is that the location of abnormal enhanced lesions was not a distinguishing factor for recurrence from benign postoperative findings. A peripheral margin following a deep margin on transplanted tissue was the most common site in both recurrent tumors and postoperative changes. Therefore, imaging findings should be carefully reviewed. Seven recurrent tumors were found at regional sites apart from the transplanted tissue. These tumors might have originated from small tumor emboli trapped within the lymphatics [18].

Our study newly revealed partitioning MRI features of locoregional recurrence, defined as well-defined borders, a nodular shape, impeded water diffusion, and intense enhancement in patients with reconstructive surgery after tumor resection. These results are not different from those of previous studies of postoperative MRI of soft tissue sarcoma resection regardless of soft tissue reconstructive surgery [4,13,19]. Almost all local recurrence was nodular (39/40, 98%) in this study. Nodularity was sensitive for predicting recurrence but was of low specificity. Importantly, about half of benign postoperative changes appeared to be nodular in nature (20/36, 56%). Depending on the case, sometimes after reconstructive surgery, the anastomosis site of the blood vessel may appear as a nodule shape around the transplanted tissue. Radiation-induced pseudotumor and focal fibrosis may show enhancement and nodularity [20]. It is a practically useful finding that most of the non-nodular lesions were postoperative changes (16/17, 94%) in our study. Local recurrences were mostly (75%) well-defined, but postoperative changes were mostly (75%) ill-defined. These findings may be due to inflammatory changes and benign soft tissue edema related to operation or radiation therapy.

Local recurrences showed common hyperintensity in T2WI and FS-T2WI and isointensity in T1WI. The signal intensities of recurrences and postoperative changes were different in incidences. The subcutaneous fat, skin, and muscles that made up the flap were well distinguished using MRI, so the composition of the transplanted tissue could easily be estimated. Muscles may show alteration in signal intensity due to postoperative edema and changes due to radiation therapy. Signal intensity of the transplanted flap tissue also varies over time [20,21]. Signal intensity of recurrent tumors is also affected by radiation therapy. Thorough evaluation of multiple MRI sequences may be helpful for detecting small lesions with similar signal intensity to the transplanted soft tissue. Contrast-enhanced MRI can improve sensitivity and confidence of readers in the detection of recurrent tumors [5,22]. However, it is common for postoperative changes also to show contrast enhancement, so the specificity of this technique is low [5,22]. In cases of reconstructive surgery, the microvascular anastomosis site of transplanted tissue may be involved in local contrast enhancement. Our study newly found that the area of reconstructive surgery can show weak contrast enhancement compared to recurrent tumors. In DWI, there was no difference between the two groups; both showed hyperintensity in both the local recurrence and the postoperative change groups. However, ADC values were significantly lower in the recurrence group. A T2 shine-through effect may be a concern. DWI with ADC was useful in patients who underwent reconstructive surgery to differentiate local recurrence from postoperative changes, similar to previous studies of soft tissue reconstructive surgery [5,13,14,23,24].

Our study had several limitations. First, this was a retrospective study, and therefore there may be selection bias. Particularly, pathologically unidentified lesions that had undergone adjuvant chemotherapy or radiation therapy were not included because they could not be determined as benign or malignant. Second, multiple lesions were selected per person, although such lesions might be related and introduce error. However, we believe that it would be useful to include all individually identified lesions rather than analysis including an arbitrarily selected lesion. Third, the number of lesions analyzed was small, and the histologic types of tumors included were diverse, preventing analysis of the characteristics of tumor histologic type. Results may also be affected by the histologic type of tumor included. Fourth, due to the small number of cases and tumor type variability, we did not present the diagnostic performance of each imaging feature that distinguishes between groups with and without recurrent tumors, as measured with variables such as odds ratio or accuracy. However, the purpose of this study was achieved by comparing and presenting the frequency of normal imaging findings that may appear after reconstructive surgery with the frequency that may appear in recurrent tumors. Finally, patients had undergone various treatment combinations including radiation therapy and chemotherapy with various time intervals between treatment and MRI.

## 5. Conclusions

In conclusion, common and partitioning MRI features of patients with local recurrence who underwent reconstructive surgery after sarcoma resection tended to be a nodular shape, well-defined margin, moderate or intense enhancement, and low ADC. Peripheral margin of flap or skin graft and hyperintensity on DWI were commonly seen in both local recurrence and postoperative changes.

## Figures and Tables

**Table 1:** MR parameters.

Parameters	Standard Sequences	DWI
FOV (mm)	80–300	80–300
Size of Matrix (mm)	128 × 61–512 × 358	98 × 84–150 × 136
TR (ms)/TE (ms)	T1WI: 700–800/11–17 T2WI: 3000–5500/53–88	3500–7500/50–63
Method of fat suppression	Dixon	SPAIR
Slice thickness (mm)	3–7	3–7
Interslice gap (mm)	0–0.6	0–0.6
TSE factor or EPI factor	T1WI: 3 T2WI: 17	31–61
NEX	1	3–8

DWI: diffusion-weighted imaging. FOV: field of view. TR: repetition Time. TE: echo Time. T1WI: T1-weighted MR imaging. T2WI: T2-weighted MR imaging. SPAIR: spectral attenuated inversion recovery. TSE: turbo spin echo. EPI: echo planar imaging. NEX: number of excitations.

**Table 2:** Patient demographics.

Variables		Number of Patients (*n* = 37)
Age (years) ^a^		60.1 ± 15.7
Sex	Male	19
	Female	18
Histopathology of primary tumor	Dedifferentiated liposarcoma	1
	Dermatofibrosarcoma protuberans	3
	Extraskeletal myxoid chondrosarcoma	1
	Melanoma	11
	Myxofibrosarcoma	6
	Myxoid liposarcoma	5
	Pseudomyogenic hemangioendothelioma	1
	Rhabdomyosarcoma	1
	Squamous cell carcinoma	3
	Undifferentiated pleomorphic sarcoma	3
	Undifferentiated spindle cell sarcoma	2

^a^ mean ± standard deviation.

**Table 3:** Characteristics of included lesions.

Variables		Recurrent Tumors (*n* = 40)	Postoperative Changes (*n* = 36)
Histopathology of primary tumor	Dedifferentiated liposarcoma	1	1
	Dermatofibrosarcoma protuberans	0	3
	Extraskeletal myxoid chondrosarcoma	0	2
	Melanoma	6	11
	Myxofibrosarcoma	10	7
	Myxoid liposarcoma	12	3
	Pseudomyogenic hemangioendothelioma	3	1
	Rhabdomyosarcoma	1	0
	Squamous cell carcinoma	2	2
	Undifferentiated pleomorphic sarcoma	5	3
	Undifferentiated spindle cell sarcoma	0	3
Anatomic site	Trunk and pelvis	6	11
	Upper arm and forearm	11	8
	Thigh and leg	6	10
	Ankle and foot	17	7
Resection margin of primary tumor excision	R1 resection (positive tumor margin)	15	9
	R0 resection (negative tumor margin)	25	27
Type of reconstructive surgery	Free flap	18	24
	Local flap	11	10
	Skin graft	11	2
Prior chemotherapy	Positive	8	8
	Negative	32	28
Prior radiation therapy	Positive	26	15
	Negative	14	21

**Table 4:** Morphology of recurrent tumor.

Variables		Local Recurrences (*n* = 40)	Postoperative Changes (*n* = 36)	*p* Values
Location on the transplanted soft tissue ^a^	Peripheral margin	25	22	0.907
	Deep margin	11	12	0.583
	Regional	7	3	0.241
Border	Well-defined	30	9	<0.001 *
	Ill-defined	10	27	
Shape	Nodular	39	20	<0.001 *
	Non-nodular	1	16	
Size (cm) ^b^		2.6 ± 2.2	1.7 ± 1.0	0.020 *

^a^ If a lesion showed multiple relations with the transplanted soft tissue margin, multiple counts were performed. ^b^ mean ± standard deviation. * statistically significant.

**Table 5:** MRI signal intensity and DWI findings for recurrent tumors and postoperative changes.

Sequences	Signal Intensity of MRI	Local Recurrences (*n* = 40)	Postoperative Changes (*n* = 36)	Hyperintensity vs. iso- or Hypointensity (*p* Value)
T1WI	Hyperintensity	12	2	0.007 *
	Isointensity	26	31	
	Hypointensity	2	3	
T2WI	Hyperintensity	34	15	<0.001 *
	Isointensity	4	17	
	Hypointensity	2	4	
FS-T2WI	Hyperintensity	38	27	0.020 *
	Isointensity	1	7	
	Hypointensity	1	2	
Contrast-enhanced FS-T1WI	Strong	20	3	<0.001 *
	Moderate	18	31	
	Mild	2	2	
DWI with high *b*-value	Hyperintensity	37	30	0.294
	Isointensity	1	4	
	Hypointensity	2	2	
ADC (μm^2^/s) ^a^		1297.0 ± 590.9	1634.6 ± 462.7	0.008 *
Impeded water diffusion	Positive	22	5	<0.001 *
	Negative	18	31	

^a^ mean ± standard deviation. * statistically significant. T1WI: T1-weighted MR imaging. T2WI: T2-weighted MR imaging. FS: fat-suppressed. DWI: diffusion-weighted imaging. ADC: apparent diffusion coefficient.

**Table 6:** Diagnostic performance of the principal MRI features suggestive of local recurrence.

MRI Features	Sensitivity	Specificity	Accuracy	Positive Predictive Value	Negative Predictive Value
Well-defined border	75.0	75.0	75.0	76.9	73.0
Nodular shape	97.5	44.4	72.4	66.1	94.1
Strong enhancement	50.0	91.7	69.7	87.0	62.3
Impeded water diffusion	55.0	86.1	69.7	81.5	63.3

Data were percents.

## Data Availability

The data presented in this study are available on request from the corresponding author.

## Abbreviations

ADC	apparent diffusion coefficient
CE	contrast-enhanced imaging
DWI	diffusion-weighted imaging
FS-T2WI	fat-suppressed T2-weighted imaging
T1WI	T1-weighted imaging
T2WI	T2-weighted imaging
